# Association of Retinopathy of Prematurity With Low Levels of Arachidonic Acid: A Secondary Analysis of a Randomized Clinical Trial

**DOI:** 10.1001/jamaophthalmol.2017.6658

**Published:** 2018-02-08

**Authors:** Chatarina A. Löfqvist, Svetlana Najm, Gunnel Hellgren, Eva Engström, Karin Sävman, Anders K. Nilsson, Mats X. Andersson, Anna-Lena Hård, Lois E. H. Smith, Ann Hellström

**Affiliations:** 1 Section of Ophthalmology, Department of Clinical Neuroscience, Institute of Neuroscience and Physiology, Sahlgrenska Academy, University of Gothenburg, Göteborg, Sweden; 2 Department of Paediatrics, Institute of Clinical Sciences, Sahlgrenska Academy, University of Gothenburg, Göteborg, Sweden; 3 Department of Biology and Environmental Sciences, University of Gothenburg, Göteborg, Sweden; 4 Department of Ophthalmology, Boston Children’s Hospital, Harvard Medical School, Boston, Massachusetts

## Abstract

**Importance:**

Mice with oxygen-induced retinopathy fed matched diets except for ω-3 long-chain polyunsaturated fatty acids (LC-PUFAs) vs ω-6 LC-PUFAs demonstrate relative antiangiogenic and neuroprotective associations of ω-3 LC-PUFAs. However, supplementing preterm infants with LC-PUFAs has been inconsistent in reducing major preterm morbidities. However, few studies measured serum lipid levels after supplementation.

**Objective:**

To examine the associated risk of retinopathy of prematurity (ROP) from the levels of circulating ω-3 and ω-6 LC-PUFAs.

**Design, Setting, and Participants:**

This longitudinal clinical study was a further analysis of serum lipid levels from a randomized controlled trial cohort of 90 infants born at gestational age (GA) less than 28 weeks. From April 4, 2013, to September 22, 2015, cord blood samples, followed by venous blood samples, were obtained at birth and at 1, 7, 14, and 28 days after birth and then at postmenstrual age (PMA) 32, 36, and 40 weeks at the neonatal intensive care unit at Sahlgrenska University Hospital in Göteborg, Sweden.

**Main Outcomes and Measures:**

Serum phospholipid fatty acids were transmethylated and measured by gas chromatography–mass spectrometry. Mann-Whitney test, logistic regression Spearman rank correlation, and receiver operating characteristic curve analysis were used to compare differences between infants with no ROP and infants who developed ROP.

**Results:**

Serum levels from 78 infants (43 male [55%]; mean [SD] GA, 25.5 [1.4] weeks) with a known ROP outcome were evaluated. Lower area under the curve (AUC) of arachidonic acid (AA) (20:4 ω-6) was seen in infants with a later diagnosis of ROP compared with infants with no ROP in the first month of life (mean, 34.05 [95% CI, 32.10-36.00] vs 37.15 [95% CI, 34.85-39.46]; *P* < .05). In addition, lower levels of AA at 32 weeks’ PMA were seen in infants with later severe ROP compared with in those without ROP (mean, 7.06 [95% CI, 6.60-7.52] vs 8.74 [95% CI, 7.80-9.67]; *P* < .001). In logistic modeling, low postnatal serum levels of AA and GA at birth identified with a sensitivity greater than 90% of infants who developed ROP.

**Conclusions and Relevance:**

Low postnatal levels of the ω-6 LC-PUFAs (AA) are strongly associated with ROP development. Evaluating postnatal AA fraction after birth in addition to GA may be useful for ROP prediction.

**Trial Registration:**

clinicaltrials.gov Identifier: NCT02760472

## Key Points

**Question:**

How are circulatory levels of ω-3 and ω-6 long-chain polyunsaturated fatty acids associated with retinopathy of prematurity?

**Findings:**

In this secondary analysis of data from a randomized clinical trial of 78 infants, higher ω-6 polyunsaturated fatty acid arachidonic acid serum levels were associated with a lower risk of developing any retinopathy of prematurity, as well as sight-threatening retinopathy of prematurity.

**Meaning:**

The ω-6 polyunsaturated fatty acid arachidonic acid is associated with a lower risk of developing retinopathy of prematurity; while these findings do not indicate a cause-and-effect relationship, they support testing the hypothesis of supplementation of arachidonic acid in extremely preterm infants to reduce the risk of developing retinopathy of prematurity.

## Teaser

This secondary analysis of a randomized clinical trial examines the predicted risk of retinopathy of prematurity from the levels of circulating ω-3 and ω-6 long-chain polyunsaturated fatty acids in neonates.

## Introduction

Retinopathy of prematurity (ROP) is a potentially sight-threatening neurovascular disease that in settings of high-quality care affects primarily extremely preterm infants. It is a condition in which a variety of factors induce a first phase of reduced retinal vessel growth and microvascular degeneration, followed by a second phase of pathologic neovascularization, which may lead to retinal detachment.^1^ Oxygen supplementation is a major risk factor for ROP, and higher oxygen saturation targets are associated with increased ROP risk.^2^ In rodents, hyperoxia^3^ and fluctuating oxygenation^4^ lead to delayed physiologic retinal vascularization and subsequent neovascularization. Control of oxygen administration has been critical to ROP prevention.^5^ However, the optimal degree of oxygenation of a very immature individual is still not known.

The challenge of neonatal intensive care is to balance between the needs of a fetus at a certain developmental stage and the conditions required to sustain growth and development during extrauterine life. When rapid brain and retina development take place during the third trimester, long-chain polyunsaturated fatty acids (LC-PUFAs), which are structural and functional components of most cell membranes, are selectively transferred from the mother to the fetus.^6,7^ Of these, docosahexaenoic acid (DHA) (22:6 ω-3) and arachidonic acid (AA) (20:4 ω-6) are the most abundant LC-PUFAs in the central nervous system.^8^ With current standard care, extremely preterm infants receive insufficient amounts of both DHA and AA in parenteral nutrition, as reflected in low serum levels of these fatty acids (FAs).^9,10,11^ Results of in vivo animal studies^12,13,14,15^ comparing diets with ω-3 vs ω-6 LC-PUFAs suggest that these essential dietary lipids affect retinal health and disease. Research on LC-PUFAs in preterm infants has focused mainly on the role of ω-3 LC-PUFAs and in particular on DHA, which is crucial for brain and eye development. In the retina, DHA is enriched in rod photoreceptor outer segments^16^ and is essential for their differentiation and survival^17^ and signal transduction.^18^ A recently published clinical trial (the Donna Mega Study^19^) comparing the effects of the parenteral emulsion SMOFlipid with 15% fish oil (Fresenius Kabi) vs Clinoleic (Baxter) on ROP and other morbidities and growth failed to find any effect on outcome measurements.^20^ In other studies performed in Europe, Turkey, and Australia, the use of dietary supplementation with ω-3 LC-PUFAs has been inconsistent in preventing ROP, with a reduction in any ROP but not a reduction in severe ROP in very preterm infants in one study^21^ and no reduction of ROP with enteral DHA supplementation^22^ or with parenteral nutrition^23^ in a second study.

Fish oil supplementation^20,21,22,23,24^ has been shown to affect serum and plasma levels^20,24^; to our knowledge, however, the influence on longitudinal levels from birth to 40 weeks’ postmenstrual age (PMA) has not been evaluated in extremely preterm infants, who are at most risk of developing severe ROP. The aim of this study was to examine the association between circulating longitudinal levels of LC-PUFAs and ROP in extremely preterm infants.

## Methods

### Study Design

The Donna Mega Study^19^ was a randomized, open-label, controlled trial conducted at a single site in Sweden designed to compare the effects of the parenteral emulsion SMOFlipid (containing 15% fish oil rich in ω-3 LC-PUFAs) with olive oil–based Clinoleic (without fish oil) on ROP and other morbidities and growth. The trial was approved by the Regional Ethical Board, Gothenburg (Dnr 303-11) at the University of Gothenburg, Göteborg, Sweden. Informed written consent was obtained for all participants from their parents or guardians. No manufacturer of the parenteral products contributed to the design of the study, the accrual or analysis of the data, or the preparation of the manuscript. The protocol for the Donna Mega Study is available online.^19^ The major outcome of evaluating an association between parenteral nutrition with and without fish oil and ROP and other morbidities and growth was described in 2017 by Najm et al.^20^

### Patients and Nutritional Management

Infants born at gestational age (GA) less than 28 weeks were enrolled from the neonatal intensive care unit at Sahlgrenska University Hospital in Göteborg, Sweden, from April 4, 2013, to September 22, 2015. Exclusion criteria were major malformations. Parents of 90 of 138 eligible infants agreed to participation after written informed consent. The nutritional strategy has been described previously.^20,25^ Briefly, all infants received parenteral and enteral nutrition according to clinical routine. Parenteral nutrition was initiated as soon as possible after birth. The parenteral lipid dosing strategy was to deliver a dose of 2 to 3 g/kg of body weight every 24 hours. Enteral nutrition used either maternal or donor breast milk with individualized fortification. Minimal enteral feeding was started within 3 hours of birth and was administered every 2 to 3 hours (1-2 mL per meal), with a gradual increase in volume. Seventy-eight infants (43 male [55%]; mean [SD] GA, 25.5 [1.4] weeks) with a known ROP outcome were evaluated.

### Eye Examinations

Retinopathy of prematurity screening started at 5 to 6 weeks’ postnatal age but not before 31 weeks’ PMA. Retinal examinations through dilated pupils were performed biweekly to twice a week depending on ROP severity. Retinopathy of prematurity was classified according to the international classification,^26^ and the recommendations of the Early Treatment for Retinopathy of Prematurity Cooperative Group^27^ were followed for treatment.

### Morbidities

Diagnoses of bronchopulmonary dysplasia, necrotizing enterocolitis, patent ductus arteriosus, and sepsis were retrieved from clinical records. Bronchopulmonary dysplasia was defined as moderate to severe lung disease with a need for oxygen supplementation at 36 weeks’ PMA, necrotizing enterocolitis was diagnosed by clinical signs and radiologic findings (Bell stages 2-3), and patent ductus arteriosus was registered when the infant had clinical symptoms that required either pharmacological or surgical treatment. Sepsis was diagnosed by clinical symptoms accompanied by a blood culture positive for bacteria or fungi. When the culture contained *Staphylococcus epidermidis*, an elevated C-reactive protein level (>20 mg/L) was required for diagnosis (to convert C-reactive protein level to nanomoles per liter, multiply by 9.524).

### Blood Sampling and Laboratory Analyses

Cord blood samples, followed by venous blood samples, were obtained at birth and at postnatal days 1, 7, 14, and 28, as well as at PMA of 32, 36, and 40 weeks. The serum phospholipids were extracted^28^ and then fractionated on a single aminopropyl cartridge (Sep-Pak; Waters Corporation). The FA methyl esters derived from the serum phospholipids were measured by gas chromatography–mass spectrometry and expressed as molar percentage.^20^ Longitudinal phospholipid LC-PUFA profiles were analyzed, and the quantified FAs are listed in the eTable in the Supplement.

### Statistical Analysis

It was previously found that supplementation with SMOFlipid containing 15% fish oil during parenteral nutrition increases eicosapentaenoic acid (EPA) substantially and DHA marginally, reduces AA, and decreases the AA to DHA ratio.^20^ As expected, the infants in the present study who developed severe ROP had younger GA (mean [SD], 24.3 [1.1] vs 26.1 [1.5] weeks) and lower birth weight (mean [SD], 693 [170] vs 1010 [192] g) compared with infants with no ROP. Therefore, data were analyzed in the following 2 time sets: (1) the first month of life, where area under the curve (AUC) was used to analyze the longitudinal data, and (2) at PMA 32, 36, and 40 weeks, where the measured level at the time point was used. This allowed for adjustment for parenteral nutrition, GA at birth, and birth weight. Severe ROP was defined as ROP stage 3 or treated ROP. Binary logistic regression models on LC-PUFA levels with *P* < .20 were used to calculate probability values for any ROP or severe ROP. Receiver operating characteristic (ROC) curve analyses then used the calculated probability value as the test variable, together with the FA variable and clinically relevant variables, such as GA, birth weight, and nutritional regimen. For comparison of frequencies, χ^2^ test was used. Statistical analysis was performed using a software program (SPSS 23 for Microsoft Windows; IBM). In all analyses, 2-sided *P* < .05 was considered significant.

## Results

### Clinical Characteristics of the Patients

Among the 90 infants recruited for the original study, 78 (87%) survived to the final examination for ROP and were included in this longitudinal clinical study. Seventeen infants did not develop ROP, 8 developed stage 1 ROP, 22 developed stage 2 ROP, and 31 infants developed stage 3 ROP. In total, 22 infants had type 1 ROP and were treated. The clinical characteristics of the study infants are listed in Table 1.

**Table 1.** Clinical Characteristics of 78 Infants Completing the Study

Variable	Value
Gestational age, mean (SD), wk	25.5 (1.4)
Birth weight, mean (SD), g	797 (223)
Weight SD score, mean (SD)	−0.82 (1.20)
Birth weight small for gestational age, No. (%)	11 (14)
Male, No. (%)	43 (55)
Bronchopulmonary dysplasia, No. (%)	39 (50)
Necrotizing enterocolitis, No. (%)	5 (6)
Patent ductus arteriosus, No. (%)	54 (69)
Sepsis, No. (%)^a^	30 (39)

^a^ Sepsis was verified by culture and/or C-reactive protein level.

### Serum Fractions of LC-PUFAs and ROP

#### First Postnatal Month

Area under the curve analysis demonstrated that during the first month of life AA was the only LC-PUFA that had significantly lower serum fractions in infants who later developed any or severe ROP compared with infants without ROP. There was no correlation between GA at birth and the first month of life AA AUC. There were no differences in any of the other ω-3 or ω-6 LC-PUFA AUCs during the first postnatal month between ROP groups (Table 2).

**Table 2.** Comparison of Monounsaturated and Polyunsaturated Fatty Acids During the First Month of Life Between Groups

Variable	Area Under the Curve, Mean (95% CI)			*P* Value^a^	
	No ROP (n = 17)	Any ROP (n = 61)	Severe ROP (n = 31)	Group 1	Group 2
**ω-3**					
18:3 ω-3 (α-Linolenic acid)	0.37 (0.30-0.44)	0.44 (0.35-0.52)	0.51 (0.36-0.66)	.43	.20
20:5 ω-3 (Eicosapentaenoic acid)	3.34 (2.74-3.95)	3.64 (3.28-4.00)	3.76 (3.22-4.29)	.43	.28
22:6 ω-3 (Docosahexaenoic acid)	10.70 (9.75-11.66)	10.17 (9.72-10.63)	10.17 (9.37-10.85)	.29	.23
**ω-6**					
18:2 ω-6 (Linoleic acid)	55.30 (52.23-58.38)	53.27 (51.40-55.14)	53.35 (50.59-56.11)	.30	.47
18:3 ω-6 (γ-Linolenic acid)	0.66 (0.55-0.77)	0.75 (0.69-0.80)	0.77 (0.68-0.86)	.18	.24
20:2 ω-6 (Eicosadienoic acid)	3.83 (3.12-4.54)	4.53 (3.98-5.08)	4.51 (3.61-5.40)	.21	.09
20:3 ω-6 (Dihomo-γ-linolenic acid)	11.33 (10.36-12.30)	10.63 (10.14-11.11)	10.23 (9.55-10.91)	.18	.08
20:4 ω-6 (Arachidonic acid)	37.15 (34.85-39.46)	33.99 (32.85-35.14)	34.05 (32.10-36.00)	.01	.04

Abbreviation: ROP, retinopathy of prematurity.

^a^ Group 1 comparison is infants with any ROP vs no ROP. Group 2 comparison is infants with severe ROP vs no ROP.

#### At 32 Weeks’ PMA

**Figure 1.**
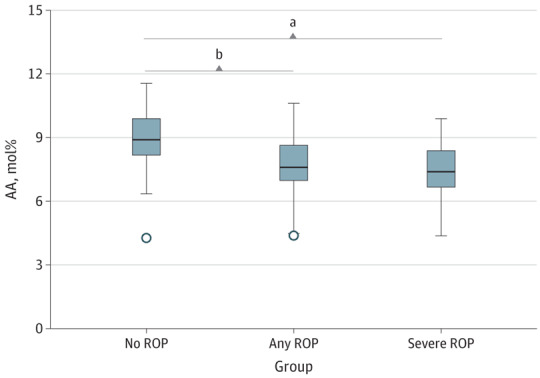
Differences in Serum Fractions of Arachidonic Acid (AA) at 32 Weeks’ Postmenstrual Age Shown are the medians (outlier 5% to maximum) for infants with no and any retinopathy of prematurity (ROP) and median (minimum to maximum) for infants with severe ROP (ROP stage 3 or T). mol% Indicates percentage of total quantified fatty acids. ^a^*P* < .01. ^b^*P* < .001.

The fraction of ω-3 LC-PUFA 18:3 ω-3 (α-linolenic acid) was significantly lower at 32 weeks’ PMA in infants with severe ROP than in infants with no ROP, but no difference in 18:3 ω-3 levels was seen between the groups with any ROP vs no ROP. Regarding the fraction of ω-3 LC-PUFAs EPA and DHA, no differences were found between infants with no ROP compared with those with any or severe ROP at this time point. At 32 weeks, the fraction of ω-6 LC-PUFA 20:3 ω-6 (dihomo-γ-linolenic acid) was significantly lower in infants with severe ROP compared with in infants with no ROP, but no difference in 20:3 ω-6 levels was seen in infants with any vs no ROP (Table 3). There was correlation between GA at birth and AA serum fractions at 32 weeks’ PMA (*r* = 0.234, *P* < .05). However, the ω-6 LC-PUFA 20:4 ω-6 (AA) fraction was lower at 32 weeks’ PMA in infants with no ROP (mean, 8.74 [95% CI, 7.80-9.67]), in infants with severe ROP (mean, 7.06 [95% CI, 6.60-7.52]), and in infants with any ROP (mean, 7.26 [95% CI, 6.91-7.60]) after adjustment for GA (Table 3 and Figure 1). No differences in AA fractions circulating in the serum were found between any of the groups with ROP vs without ROP at 36 weeks’ and 40 weeks’ PMA.

**Table 3.** Summary of Median (mol%) Differences at 32 Weeks’ Postmenstrual Age (PMA) Between Study Groups

Variable	*P* Value for Comparison^a^	
	Any ROP vs No ROP	Severe ROP vs No ROP
**ω-3**		
18:3 ω-3 (α-Linolenic acid)	.08 (↑)	.01 (↑)
20:5 ω-3 (Eicosapentaenoic acid)	.38	.64
22:6 ω-3 (Docosahexaenoic acid)	.40	.19
**ω-6**		
18:2 ω-6 (Linoleic acid)	.96	.49
18:3 ω-6 (γ-Linolenic acid)	.90	.55
20:2 ω-6 (Eicosadienoic acid)	.93	.50
20:3 ω-6 (Dihomo-γ-linolenic acid)	.27	.048 (↓)
20:4 ω-6 (Arachidonic acid)	.003 (↓)	< .001 (↓)

Abbreviations: mol%, percentage of total quantified fatty acids; ROP, retinopathy of prematurity; (↑), higher levels in infants with ROP; (↓), lower levels in infants with ROP.

^a^ Directionality of value is indicated in parentheses.

#### Prediction of ROP

**Figure 2.**
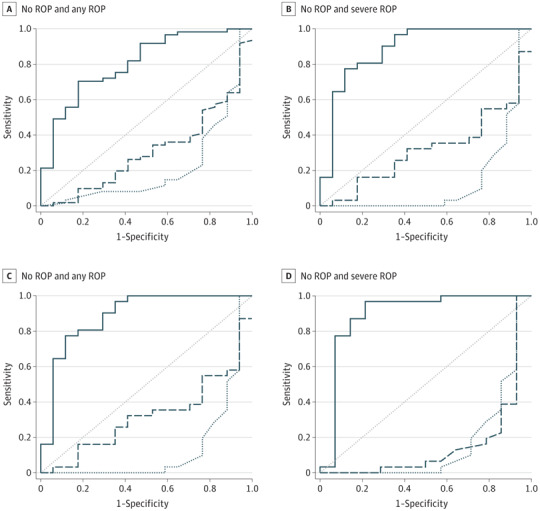
Receiver Operating Characteristic (ROC) Curve Analysis A and B, During the first month of life. C and D, At 32 weeks’ postmenstrual age. The solid line indicates the interaction between arachidonic acid and gestational age, the dashed line indicates gestational age, and the butted and dotted line indicates arachidonic acid. ROP indicates retinopathy of prematurity.

The probability of developing either any or severe ROP was affected by (1) GA at birth of the infant and (2) the AUC AA level during the first month of life, as demonstrated by logistic regression analysis. Receiver operating characteristic curve analysis revealed that the combination of circulating fraction of AA in the serum and GA can differentiate between infants without and with any ROP, with an AUC of 0.806, a sensitivity of 95.1%, and a specificity of 41.2%. An even stronger differentiation was found between infants without any ROP and infants with severe ROP, with an AUC of 0.886, a sensitivity of 90.3%, and a specificity of 70.6% (Figure 2A and B).

In addition, ROC curve analysis for the panel of AA at 32 weeks’ PMA and GA at birth had excellent predictive ability to differentiate between infants with no ROP and infants with later ROP, with an AUC of 0.825, a sensitivity of 96.6%, and a specificity of 53.3%. It had close to excellent prognostic ability to differentiate between infants with no ROP and infants with later severe ROP, with an AUC of 0.894, a sensitivity of 96.8%, and a specificity of 78.6% (Figure 2C and D).

## Discussion

We found that low postnatal serum phospholipid levels containing ω-6 LC-PUFA AA (20:4 ω-6) were strongly associated with and highly predictive of later ROP (any ROP and severe ROP). To the best of our knowledge, this finding has not been reported previously. Low levels of serum fractions of AA in the first month of life have been found in very preterm infants with late onset of sepsis.^11^ Serum fractions of AA decreased significantly after birth and remained significantly lower throughout the postnatal course in infants with ROP compared with infants with no ROP. Overall, our analysis showed that 3 of the 8 analyzed LC-PUFAs were associated with ROP. Together with LC-PUFAs, fractions of AA and the monounsaturated FAs 18:3 ω-3 (α-linolenic acid) and 20:3 ω-6 (dihomo-γ-linolenic acid) were higher in the group of infants without ROP than in those with severe ROP. Of these FAs, the most predictive LC-PUFA in logistic modeling was AA, even after adjusting for GA at birth.

Both DHA and AA are selectively transferred from the mother to the fetus during the third trimester. The relative proportion of AA in fetal blood is 2-fold higher than in maternal blood during this trimester, and fetal DHA level increases above maternal levels after approximately 30 weeks of gestation.^26^ Although DHA is the most abundant FA in cell membrane lipids in the brain and retina, AA is a dominant FA in membranes of the vascular endothelium.^27^ In brain and retina, AA is the predominant LC-PUFA up to approximately 37 and 32 gestational weeks, respectively.^29^

A large proportion (28% [22 of 78]) of the infants in this study were treated for ROP compared with infants born between 2008 and 2009 who participated in a Swedish national population-based study,^30^ where 12.8% of infants born with GA less than 28 weeks were treated. There are several possible explanations for this discrepancy. The present study was not population based, and only 90 of 138 eligible infants were enrolled, of which 78 survived. In addition, an increase in ROP treatment has been seen in Sweden, with large regional variations after implementation in 2014 of higher oxygen saturation targets.^31^

Many studies have focused on DHA and its importance for vision and cognitive development, but few studies have addressed the role of AA during fetal and neonatal life and after preterm birth. Like DHA, AA is an important component of cell membranes, where a change in composition results in changed function.^32^ Arachidonic acid is particularly enriched in the vasculature, and its metabolites can both stimulate and inhibit angiogenesis and inflammation.^33^ In addition, AA derivatives stimulate both relaxation and contraction of blood vessels,^33^ and AA metabolites contribute to neurovascular coupling in the retina.^34^

During fetal development, AA has been related to growth,^35^ and birth weight is correlated with the plasma triglyceride content of AA.^36^ Furthermore, astrocytes release AA products with proangiogenic effects on cerebral and retinal microvascular endothelial cells in culture.^37,38^ Therefore, AA is an important precursor of factors involved in angiogenesis.

Our finding of an association between low serum fractions of AA and ROP indicates that low fractions of AA in the serum may be involved in the pathogenesis of ROP. This result is in accord with work by Crawford et al,^27^ who proposed that an important component of preterm morbidities, such as ROP, is impaired vascular integrity resulting from a lack of DHA and AA in endothelial membranes.

Fish oil–based lipid solutions containing EPA may increase plasma EPA concentrations to nonphysiologic levels and decrease AA concentrations.^20,22,39^ Therefore, the use of fish oil in preterm infants without supplementation of extra AA is questionable. Reports indicate that early gavage feeding of oil with DHA from algae^40^ or low-EPA fish oil–derived DHA^41^ and algae-derived DHA plus AA from fungi^42^ successfully raises blood concentrations of these FAs, without adverse effects.

We found no association between DHA and ROP in this study. However, considering the importance of DHA for retinal development, DHA deficiency likely alters retinal development throughout the perinatal period. Docosahexaenoic acid promotes photoreceptor differentiation, protects cells from apoptosis, and is essential for photoreceptor function.^17^ In addition, retinal ganglion cells have a high DHA uptake and appear to be especially vulnerable to DHA deficiency.^43^

### Limitations

This was a small clinical study, in which we do not know to what extent the FA provided by the parenteral supplementation is absorbed. Therefore, we can only speculate that low levels of AA may be involved in the pathogenesis of ROP.

## Conclusions

After birth, extremely preterm infants in this study had a deficit of AA, which was associated with later ROP development. Therefore, the role of AA in ROP development and prediction, as well as AA supplementation, for preterm infants needs further study.

## Notes

Supplement. **eTable.** FAs Included in LC-PUFA Profiles Analyzed
