# Supplementary material for: Association of Retinopathy of Prematurity With Low Levels of Arachidonic Acid: A Secondary Analysis of a Randomized Clinical Trial
Source: JAMA Ophthalmol. 2018 Feb 8;136(3):271–7. doi: 10.1001/jamaophthalmol.2017.6658 (PMC5885898; doi:10.1001/jamaophthalmol.2017.6658)
Supplement: Supplement. — eTable. FAs Included in LC-PUFA Profiles Analyzed [file jamaophthalmol-e176658-s001.pdf]

Supplementary Online Content

Löfqvist CA, Najm S, Hellgren G, et al. Association of retinopathy of prematurity with low levels of arachidonic acid: a secondary analysis of a randomized clinical trial. *JAMA Ophthalmol*. Published online February 8, 2018. doi:10.1001/jamaophthalmol.2017.6658

eTable. FAs Included in LC-PUFA Profiles Analyzed

This supplementary material has been provided by the authors to give readers additional information about their work.

© 2018 Löfqvist CA, et al. *JAMA Ophthalmology*.

eTable. FAs Included in LC-PUFA Profiles Analyzed

**FATTY ACIDS**<br>**Common name**	Median (Min-Max) mol%
**Saturated Fatty Acids**	
14:0 (Myristic acid)	0,5 (0,3-1,2)
15:0 (Pentadecylic acid)	0,2 (0,1-0,3)
16:0 (Palmitic acid)	34,5 (30,5-41,6)
17:0 (Margaric acid)	0,3 (0,2-0,5)
18:0 (Stearic acid)	13,7 (11,1-18,0)
20:0 (Arachidic acid)	0,6 (0,1-2,0)
22: 0 (Behenic acid)	0,9 (0,5-1,5)
23:0 (Tricosylic acid)	0,2 (0,0-4,9)
24:0 (Lignoceric acid)	1,0 (0,4-2,1)
**Unsaturated Fatty Acids**	
ω **-3**	
18:3 ω-3 (α-Linolenic acid)	0,0 (0,0-3,0)
20:5 ω-3 (Eicosapentaenoic acid)	0,6 (0,2-1,6)
22:6 ω-3 (Docosahexaenoic acid)	3,4 (0,8-5,8)
ω **-6**	
18:2 ω-6 (Linoleic acid)	6,6 (3,1-12,0)
18:3 ω-6 (γ-linolenic acid)	0,1 (0,0-0,6)
20:2 ω-6 (Eicosadienoic acid)	0,6 (0,2-2,8)
20:3 ω-6 (Dihomo-γ-linolenic acid)	2,7 (1,5-4,9)
20:4 ω-6 (Arachidonic acid)	15,4 (10,2-21,7)
ω **-7**	
16:1 ω-7 Palmitoleic acid	1,0 (0,3-3,0)
18:1 ω-7 (cis-Vaccenic acid)	3,0 (1,9-4,6)
ω **-9**	
18:1 ω-9 (Oleic acid)	11,4 (7,2-15,6)
24:1 ω-9 (Nervonic acid)	2,6 (1,2-4,9)

© 2018 Löfqvist CA, et al. *JAMA Ophthalmology*.
